# Supplementary material for: An integrative analysis of the age-associated multi-omic landscape across cancers
Source: Nat Commun. 2021 Apr 20;12:2345. doi: 10.1038/s41467-021-22560-y (PMC8058097; doi:10.1038/s41467-021-22560-y)
Supplement: Supplementary file 1 — Supplementary Information [file 41467_2021_22560_MOESM1_ESM.pdf]

Supplementary Figures

An Integrative Analysis of the Age-Associated Multi-Omic Landscape across Cancers

Chatsirisupachai et al.

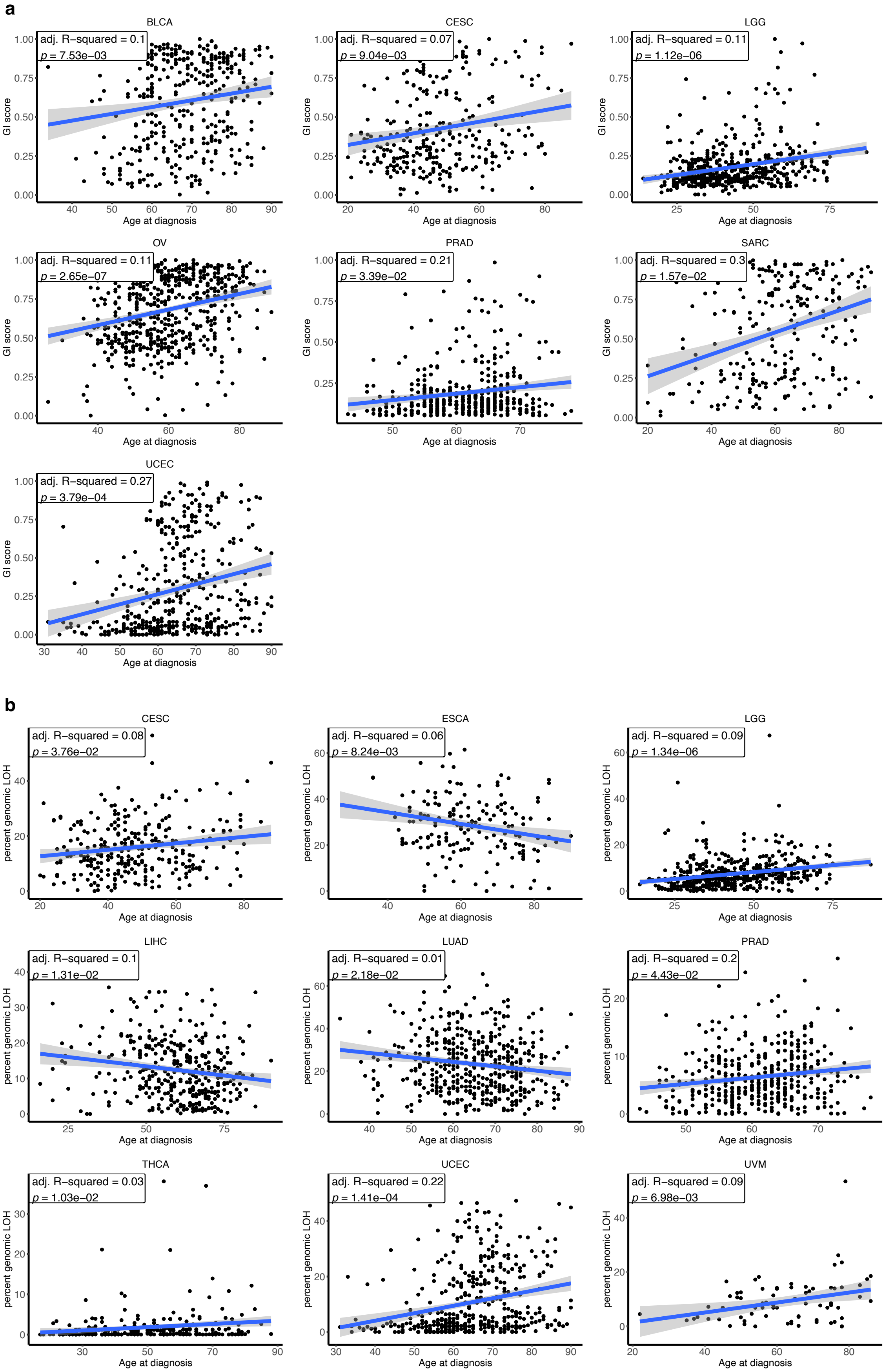

a
BLCA
adj. R−squared = 0.1
p = 7.53e−03
CESC
adj. R−squared = 0.07
p = 9.04e−03
LGG
adj. R−squared = 0.11
p = 1.12e−06
OV
adj. R−squared = 0.11
p = 2.65e−07
PRAD
adj. R−squared = 0.21
p = 3.39e−02
SARC
adj. R−squared = 0.3
p = 1.57e−02
UCEC
adj. R−squared = 0.27
p = 3.79e−04
GI score
Age at diagnosis
b
CESC
adj. R−squared = 0.08
p = 3.76e−02
ESCA
adj. R−squared = 0.06
p = 8.24e−03
LGG
adj. R−squared = 0.09
p = 1.34e−06
LIHC
adj. R−squared = 0.1
p = 1.31e−02
LUAD
adj. R−squared = 0.01
p = 2.18e−02
PRAD
adj. R−squared = 0.2
p = 4.43e−02
THCA
adj. R−squared = 0.03
p = 1.03e−02
UCEC
adj. R−squared = 0.22
p = 1.41e−04
UVM
adj. R−squared = 0.09
p = 6.98e−03
percent genomic LOH
Age at diagnosis

Supplementary Fig. 1

Association between age and (**a**) GI score and (**b**) percent genomic LOH. Multiple linear regression was performed to identify the relationship between age and GI score or percent genomic LOH for each cancer type. Only cancer types with a significant association (adj. *p*-value < 0.05) are shown together with adjusted R-squared and *p*-values before multiple hypothesis testing correction from multiple linear regression analysis. Blue lines represent best linear fit. Grey envelope denotes 95% confidence interval.

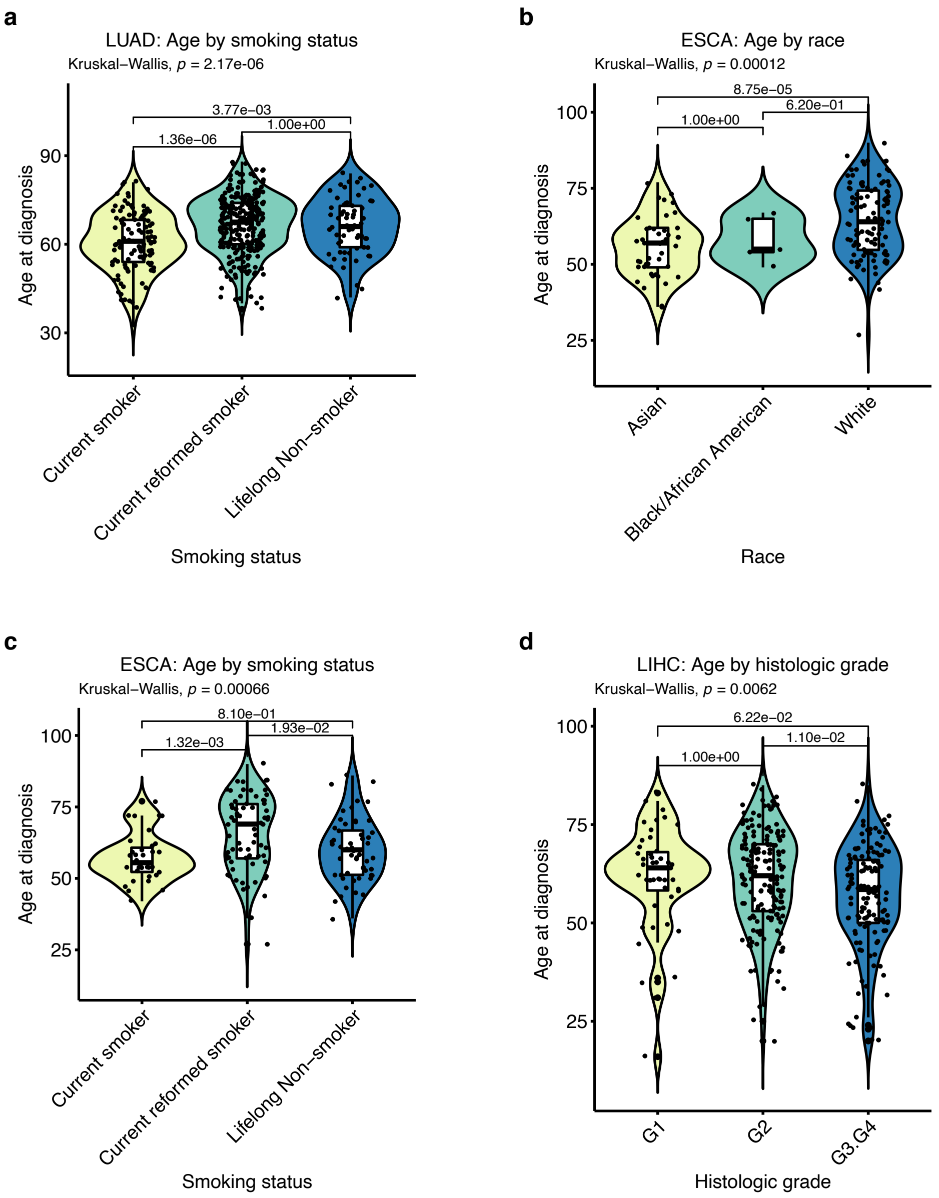

Supplementary Fig. 2

Age distribution of patients presented with different clinical factors. **a** Age distribution of lung adenocarcinoma patients by smoking status. Current smoker $n = 112$, Current reformed smoker $n = 276$, Lifelong Non-smoker $n = 62$ samples. **b** Age distribution of oesophageal cancer patients by race. Asian $n = 44$, Black/African American $n = 5$, White $n = 108$ samples. **c** Age distribution of oesophageal cancer patients by smoking status. Current smoker $n = 34$, Current reformed smoker $n = 70$, Lifelong Non-smoker $n = 54$ samples. **d** Age distribution of liver cancer patients by histologic grade. G1 $n = 46$, G2 $n = 170$, G3.G4 $n = 134$ samples. The group comparison was performed by the Kruskal-Wallis test. The pairwise comparisons were done using two-sided Dunn's test. The middle bar of the boxplot is the median. The box represents interquartile range (IQR), 25th to 75th percentile. Whiskers represent a distance of 1.5 x IQR.

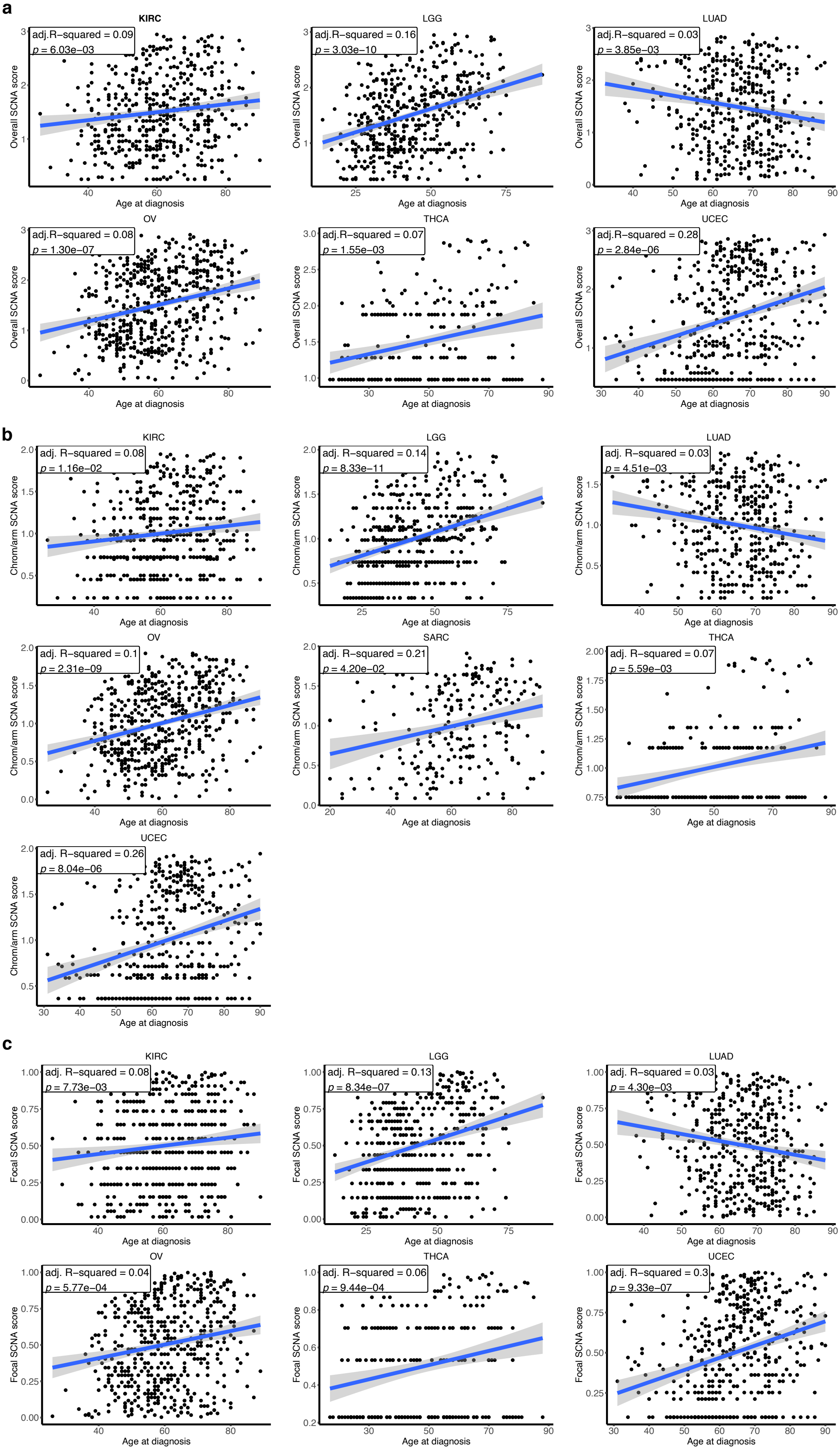
a
KIRC
adj.R−squared = 0.09
p = 6.03e−03
LGG
adj.R−squared = 0.16
p = 3.03e−10
LUAD
adj.R−squared = 0.03
p = 3.85e−03
OV
adj.R−squared = 0.08
p = 1.30e−07
THCA
adj.R−squared = 0.07
p = 1.55e−03
UCEC
adj.R−squared = 0.28
p = 2.84e−06
Overall SCNA score
Age at diagnosis
b
KIRC
adj. R−squared = 0.08
p = 1.16e−02
LGG
adj. R−squared = 0.14
p = 8.33e−11
LUAD
adj. R−squared = 0.03
p = 4.51e−03
OV
adj. R−squared = 0.1
p = 2.31e−09
SARC
adj. R−squared = 0.21
p = 4.20e−02
THCA
adj. R−squared = 0.07
p = 5.59e−03
UCEC
adj. R−squared = 0.26
p = 8.04e−06
Chrom/arm SCNA score
Age at diagnosis
c
KIRC
adj. R−squared = 0.08
p = 7.73e−03
LGG
adj. R−squared = 0.13
p = 8.34e−07
LUAD
adj. R−squared = 0.03
p = 4.30e−03
OV
adj. R−squared = 0.04
p = 5.77e−04
THCA
adj. R−squared = 0.06
p = 9.44e−04
UCEC
adj. R−squared = 0.3
p = 9.33e−07
Focal SCNA score
Age at diagnosis

Supplementary Fig. 3

Association between age and (**a**) overall SCNA score, (**b**) chromosome/arm-level SCNA score, and (**c**) focal-level SCNA score. Multiple linear regression was performed to identify the relationship between age and SCNA score for each cancer type. Only cancer types with a significant association (adj. p-value < 0.05) are shown together with adjusted R-squared and p-value before multiple hypothesis testing correction from multiple linear regression analysis. Blue lines represent best linear fit. Grey envelope denotes 95% confidence interval.

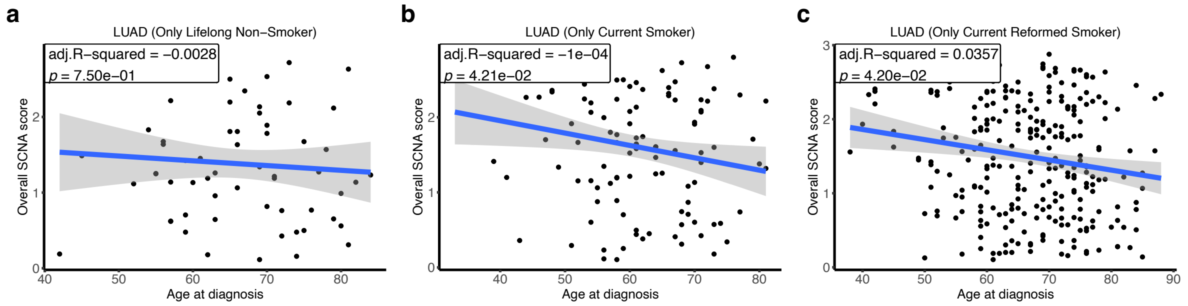

Supplementary Fig. 4

Association between age and overall SCNA score in lung adenocarcinoma separated by smoking status - (**a**) lifelong non-smoker, (**b**) current smoker and (**c**) current reformed smoker. Adjusted R-squared and *p*-value before multiple hypothesis testing correction from multiple linear regression analysis are presented. Blue lines represent best linear fit. Grey envelope denotes 95% confidence interval.

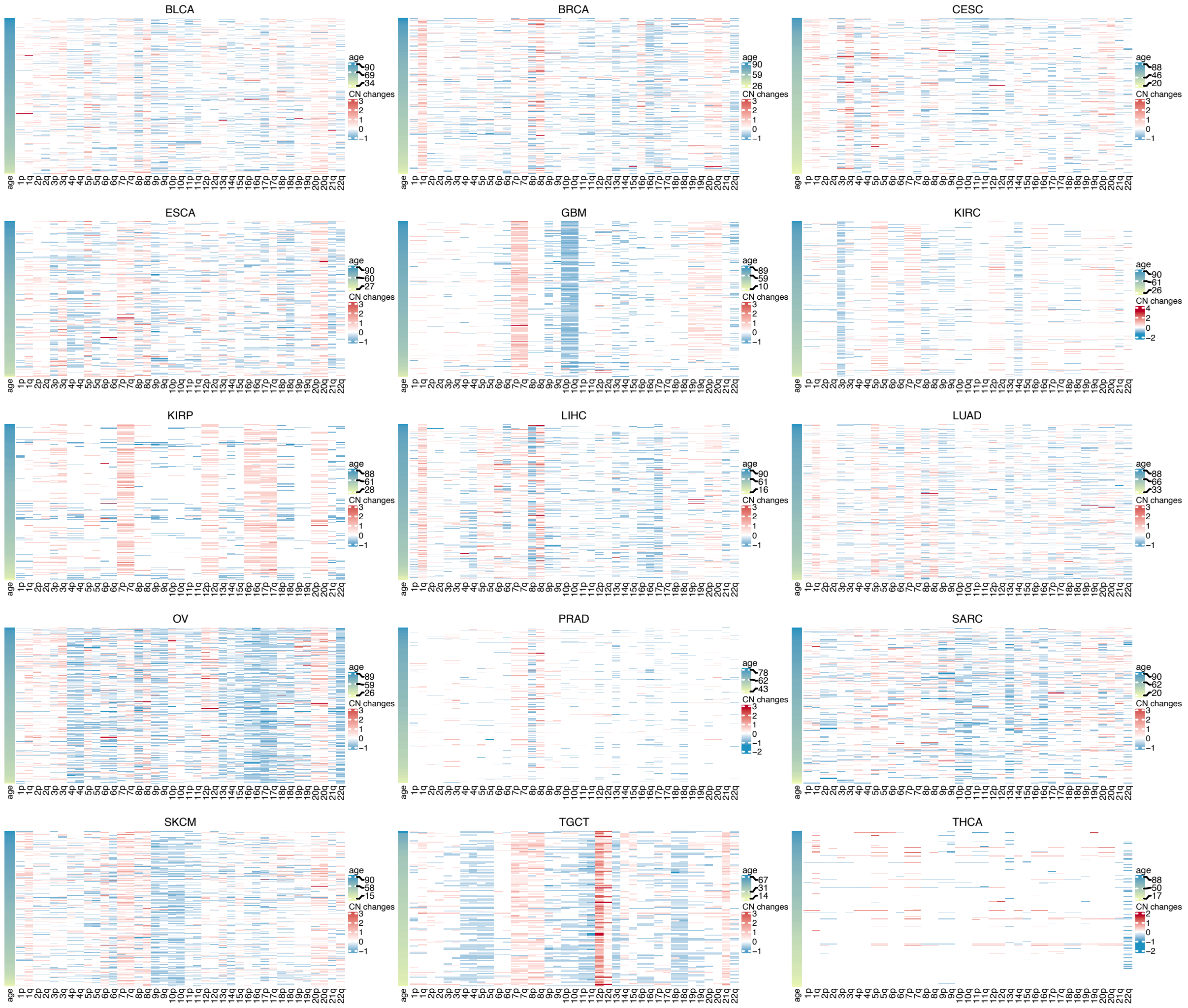

Supplementary Fig. 5

Heatmaps represent arm-level copy-number alterations across cancer types. Samples are sorted by age. Colours represent copy-number changes from GISTIC2.0, blue denotes copy-number loss and red corresponds to copy-number gain.

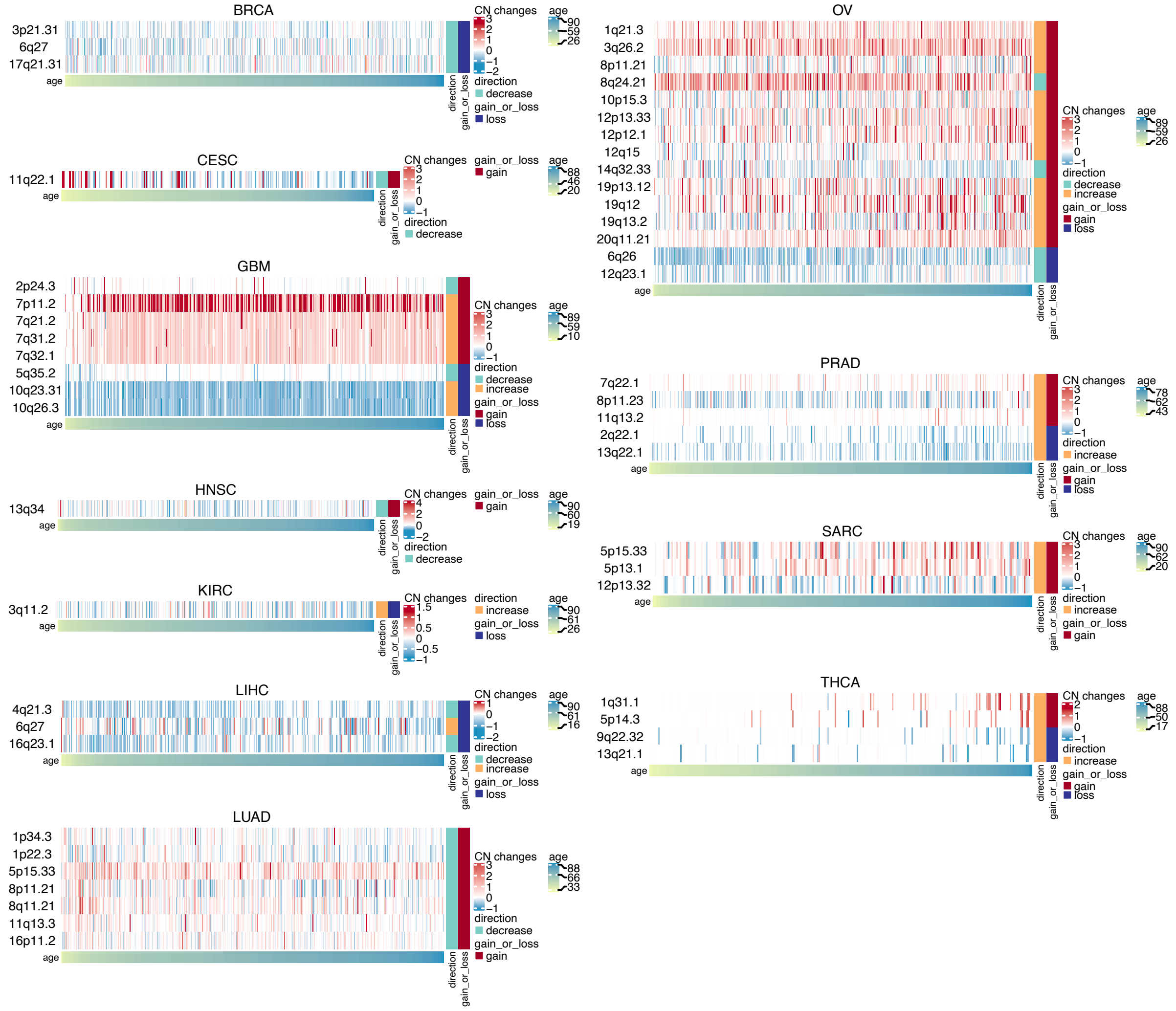

Supplementary Fig. 6

Heatmaps represent recurrent gain and loss of focal-regions that showed the age-associated patterns across cancer types. Samples are sorted by age. Colours represent copy-number changes from GISTIC2.0, blue denotes copy-number loss and red corresponds to copy-number gain. The direction legend shows whether the gain/loss of the region increases or decreases with age.

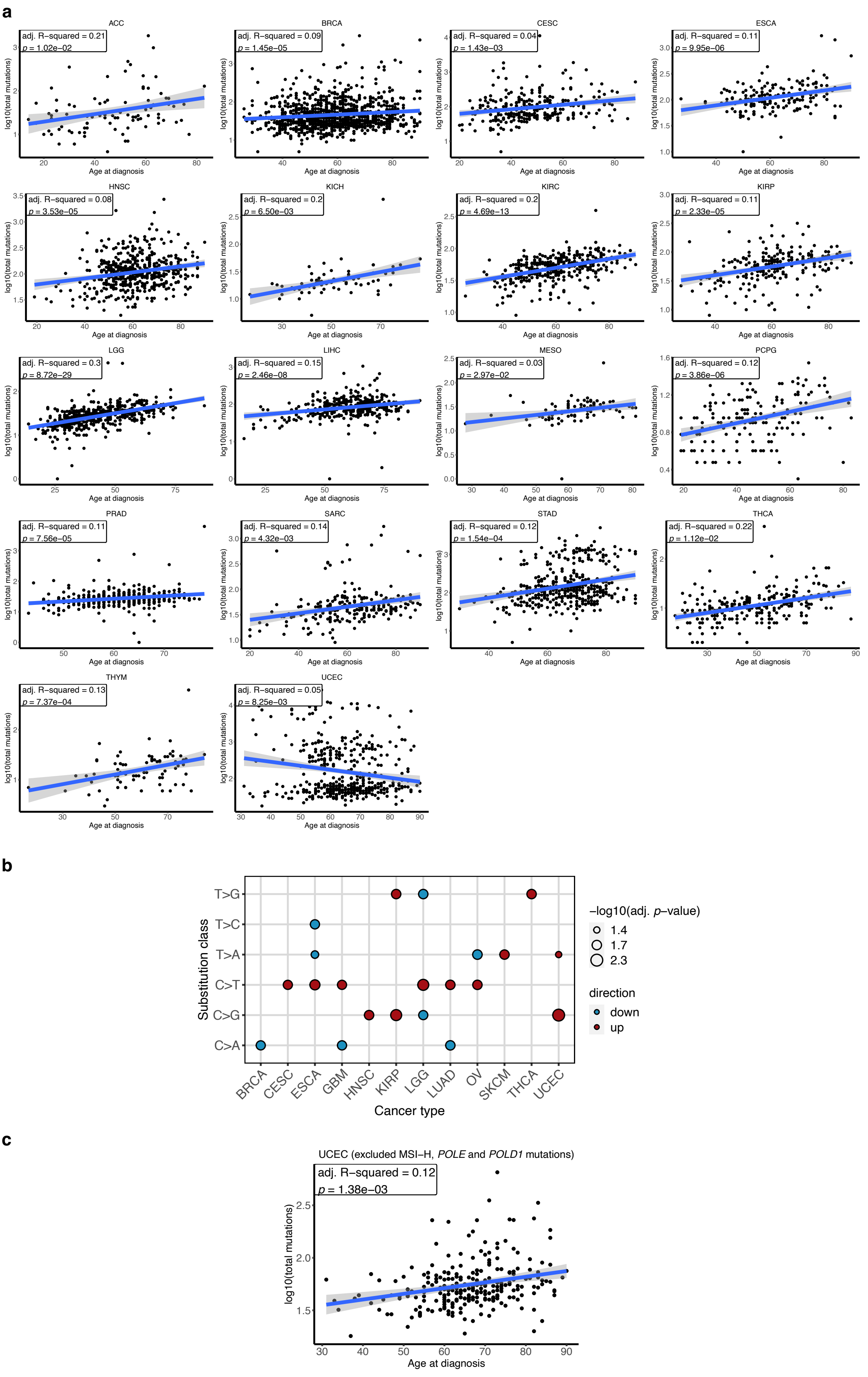

a
ACC
adj. R-squared = 0.21
p = 1.02e−02
BRCA
adj. R-squared = 0.09
p = 1.45e−05
CESC
adj. R-squared = 0.04
p = 1.43e−03
ESCA
adj. R-squared = 0.11
p = 9.95e−06
HNSC
adj. R-squared = 0.08
p = 3.53e−05
KICH
adj. R-squared = 0.2
p = 6.50e−03
KIRC
adj. R-squared = 0.2
p = 4.69e−13
KIRP
adj. R-squared = 0.11
p = 2.33e−05
LGG
adj. R-squared = 0.3
p = 8.72e−29
LIHC
adj. R-squared = 0.15
p = 2.46e−08
MESO
adj. R-squared = 0.03
p = 2.97e−02
PCPG
adj. R-squared = 0.12
p = 3.86e−06
PRAD
adj. R-squared = 0.11
p = 7.56e−05
SARC
adj. R-squared = 0.14
p = 4.32e−03
STAD
adj. R-squared = 0.12
p = 1.54e−04
THCA
adj. R-squared = 0.22
p = 1.12e−02
THYM
adj. R-squared = 0.13
p = 7.37e−04
UCEC
adj. R-squared = 0.05
p = 8.25e−03
log10(total mutations)
Age at diagnosis
b
Substitution class
T>G
T>C
T>A
C>T
C>G
C>A
BRCA
CESC
ESCA
GBM
HNSC
KIRP
LGG
LUAD
OV
SKCM
THCA
UCEC
Cancer type
−log10(adj. p−value)
1.4
1.7
2.3
direction
down
up
c
UCEC (excluded MSI−H, POLE and POLD1 mutations)
adj. R-squared = 0.12
p = 1.38e−03
log10(total mutations)
Age at diagnosis

Supplementary Fig. 7

Association between age and somatic mutations. **a** Association between age and mutation burden. Simple linear regression was performed to investigate the association between age and mutation burden. Cancer types with a significant association (adj. *p*-value < 0.05) were further investigated using multiple linear regression. The figure shows cancer type-specific analysis for every cancer that had a significant association in the simple linear regression analysis. Adjusted R-squared and *p*-value before multiple hypothesis testing correction from multiple linear regression analysis are displayed. Blue lines represent best linear fit. Grey envelope denotes 95% confidence interval. **b** Association between age and fraction contribution of each substitution class. For each substitution class in each cancer, simple linear regression was performed to investigate the association between age and fraction contribution. Cancer types with a significant association (adj. *p*-value < 0.05) were further investigated using multiple linear regression. Multiple hypothesis testing correction was done using Benjamini–Hochberg procedure. Only significant associations from multiple linear regression analysis were plotted. Circle size corresponds to the significant level, red and blue represent positive and negative associations, respectively. **c** Association between age and mutation burden in endometrial cancer after excluding tumours with MSI-H and *POLE*/*POLD1* mutations. Adjusted R-squared and *p*-value before multiple hypothesis testing correction from multiple linear regression analysis are displayed. Blue line represents best linear fit. Grey envelope denotes 95% confidence interval.

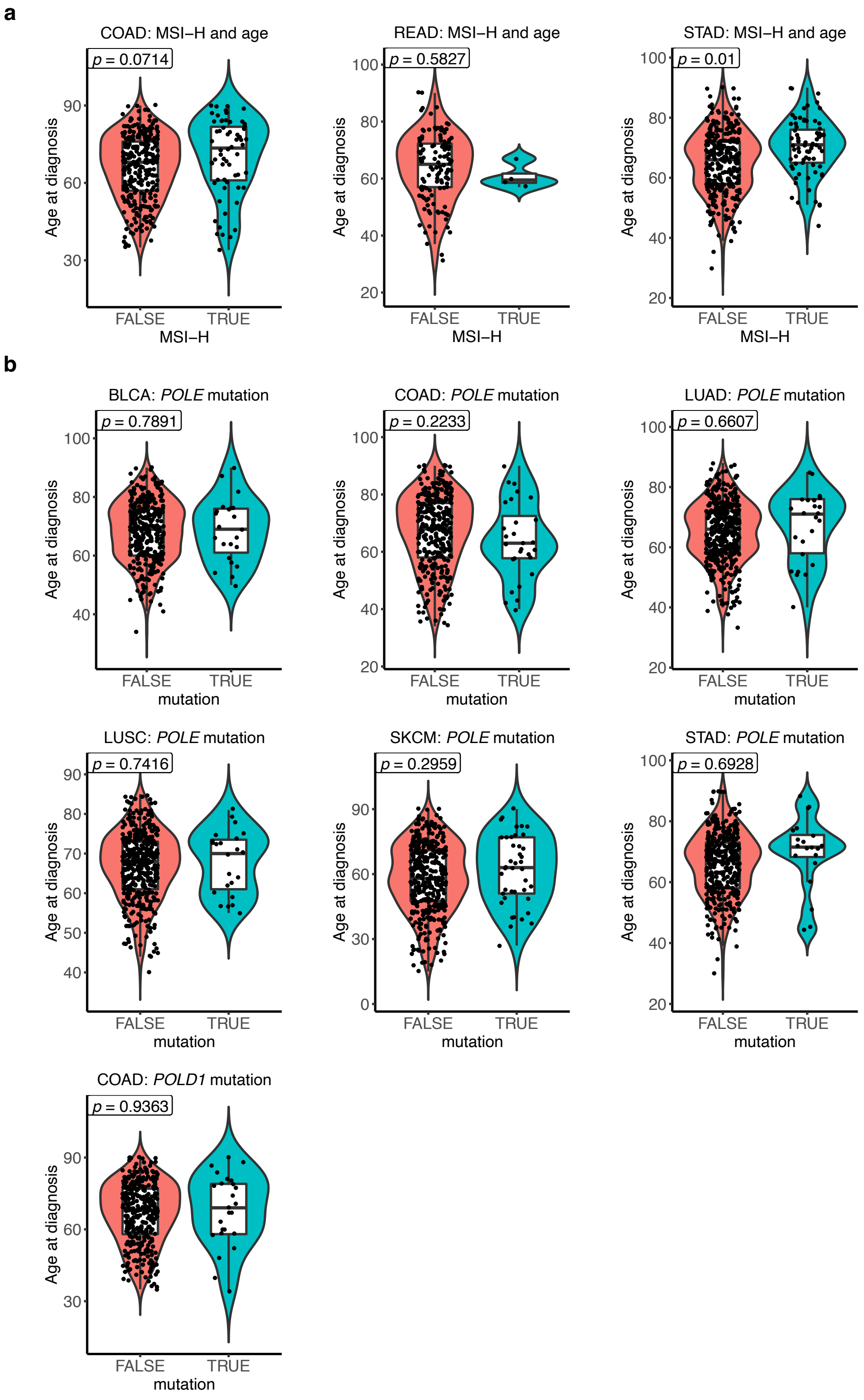

a
COAD: MSI−H and age
p = 0.0714
READ: MSI−H and age
p = 0.5827
STAD: MSI−H and age
p = 0.01
Age at diagnosis
FALSE
TRUE
MSI−H
b
BLCA: POLE mutation
p = 0.7891
COAD: POLE mutation
p = 0.2233
LUAD: POLE mutation
p = 0.6607
LUSC: POLE mutation
p = 0.7416
SKCM: POLE mutation
p = 0.2959
STAD: POLE mutation
p = 0.6928
COAD: POLD1 mutation
p = 0.9363
Age at diagnosis
FALSE
TRUE
mutation

Supplementary Fig. 8

Association between age and (**a**) MSI-H in COAD (FALSE *n* = 294, TRUE *n* = 62 samples), READ (FALSE *n* = 120, TRUE *n* = 4 samples), and STAD (FALSE *n* = 311, TRUE *n* = 74 samples), and (**b**) *POLE*/*POLD1* mutations in cancer types containing the mutations in these genes in more than 5% of the samples. BLCA:*POLE* FALSE *n* = 346, TRUE *n* = 23 samples; COAD:*POLE* FALSE *n* = 346, TRUE *n* = 28 samples; LUAD:*POLE* FALSE *n* = 431, TRUE *n* = 25 samples; LUSC:*POLE* FALSE *n* = 421, TRUE *n* = 23 samples; SKCM:*POLE* FALSE *n* = 391, TRUE *n* = 41 samples; STAD:*POLE* FALSE *n* = 365, TRUE *n* = 20 samples; COAD:*POLD1* FALSE *n* = 349, TRUE *n* = 25 samples. The statistical significance (*p*-values before multiple hypothesis testing correction are shown) was calculated from the multiple logistic regression adjusting for clinical variables. The middle bar of the boxplot is the median. The box represents interquartile range (IQR), 25th to 75th percentile. Whiskers represent a distance of 1.5 x IQR.

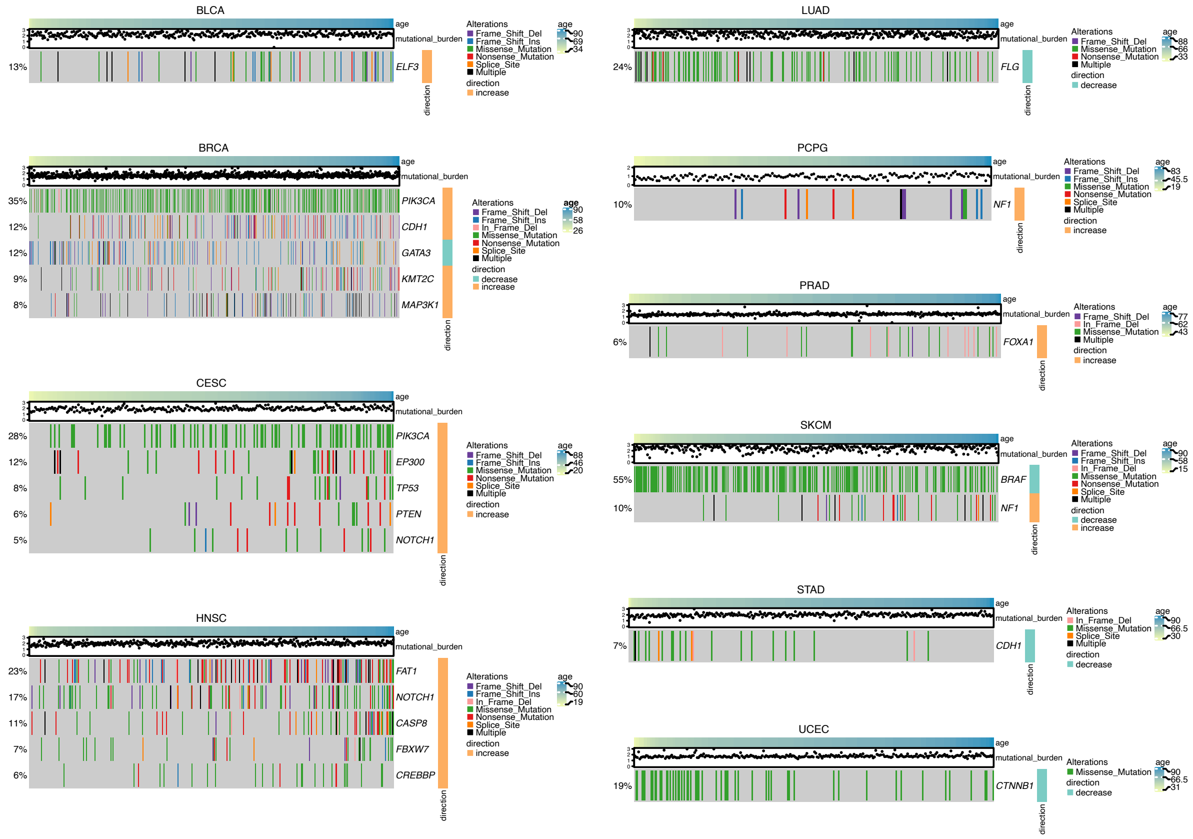

Supplementary Fig. 9

Heatmap showing age-associated mutations in 10 cancer types. Samples are sorted by age. Colours represent types of mutation. The right annotation legend indicates the direction of change, increase or decrease mutations with age. The mutation burden of samples is presented in dots above the heatmap in a log10 scale.

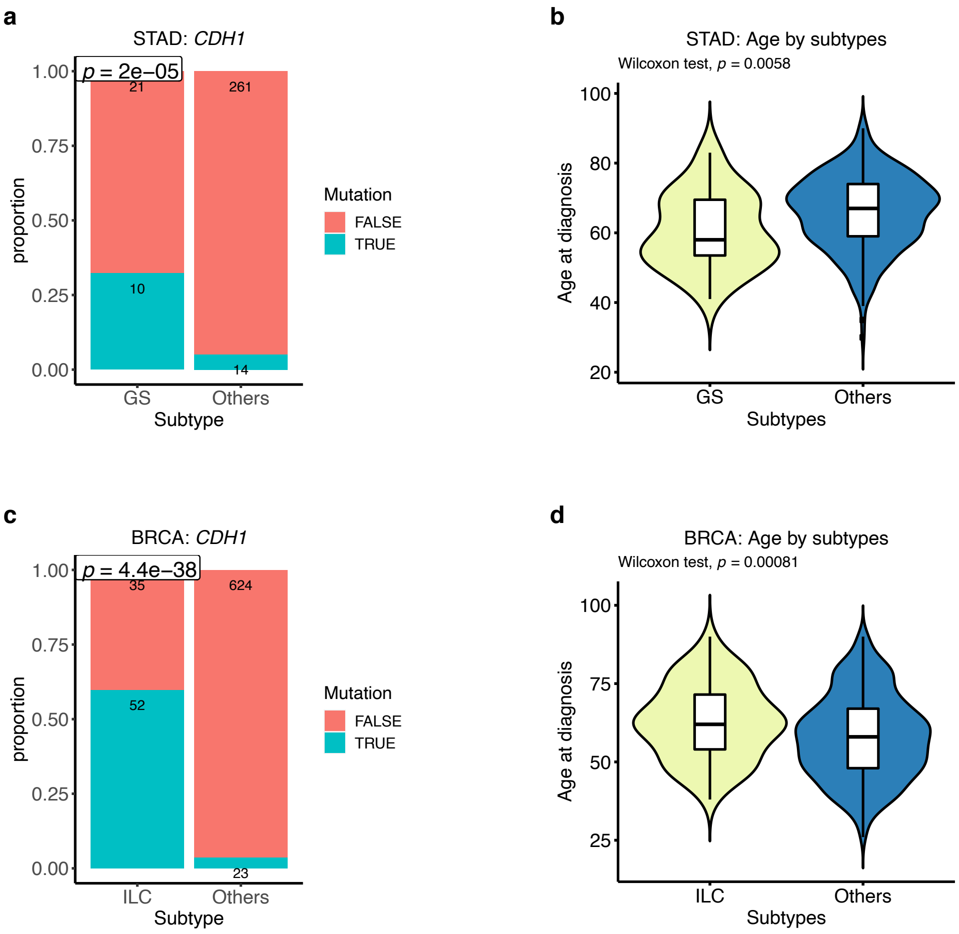

Supplementary Fig. 10

Mutations in *CDH1* by cancer subtypes. **a** Association between *CDH1* mutations and stomach cancer subtype comparing between genomically stable (GS) and other subtypes. The statistical significance (*p*-value shown) was calculated from two-sided Fisher's exact test. **b** Age distribution of genomically stable stomach cancer subtype and other stomach cancer subtypes. GS $n = 31$, Others $n = 275$ samples. The statistical significance (*p*-value shown) was calculated from two-sided Wilcoxon rank sum test. The middle bar of the boxplot is the median. The box represents interquartile range (IQR), 25th to 75th percentile. Whiskers represent a distance of 1.5 x IQR. **c** Association between *CDH1* mutations and breast cancer subtype comparing between invasive lobular carcinoma (ILC) and other subtypes. The statistical significance (*p*-value shown) was calculated from two-sided Fisher's exact test. **d** Age distribution of genomically stable stomach cancer subtype and other stomach cancer subtypes. ILC $n = 647$, Others $n = 87$ samples. The statistical significance (*p*-value shown) was calculated from two-sided Wilcoxon rank sum test. The middle bar of the boxplot is the median. The box represents interquartile range (IQR), 25th to 75th percentile. Whiskers represent a distance of 1.5 x IQR.

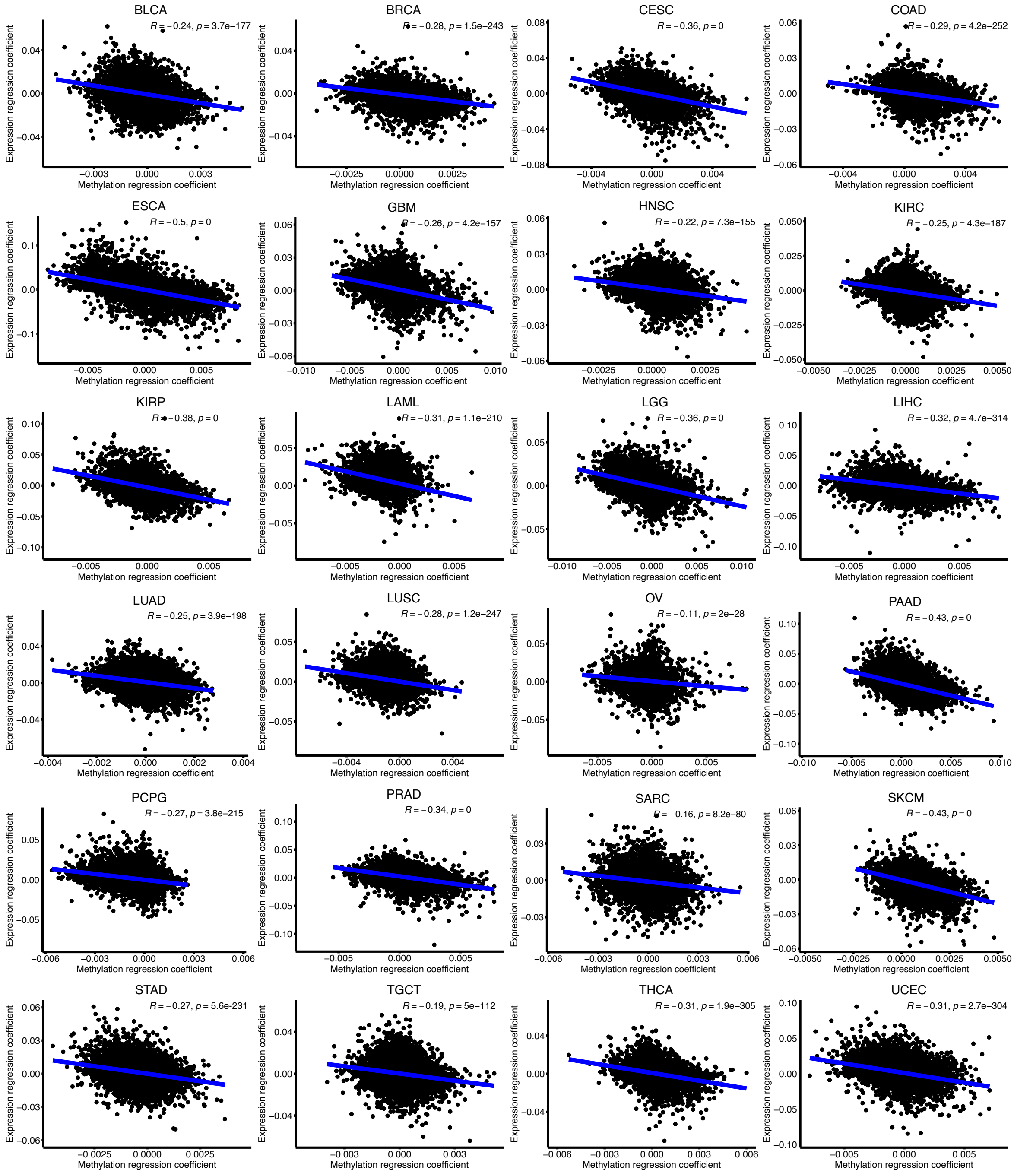

Supplementary Fig. 11

Pearson correlation between linear regression coefficient of age on DNA methylation level and linear regression coefficient of age on gene expression. The regression coefficients were obtained from the multiple linear regression analysis to investigate the association between age and DNA methylation or gene expression. Pearson correlation coefficient r and *p*-values (two-sided test) are shown in the figures.

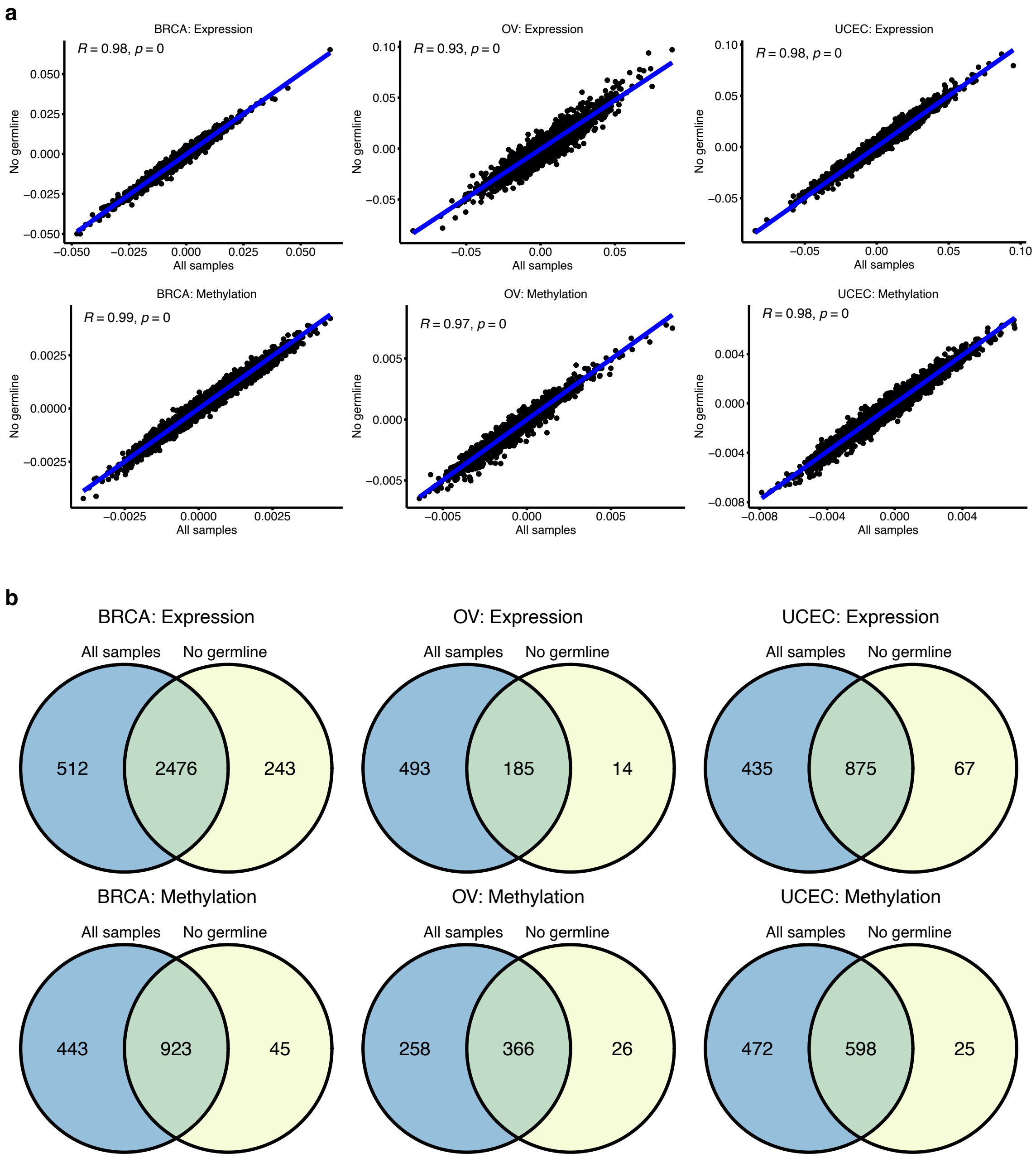

Supplementary Fig. 12

Impact of germline predisposition mutations on age-associated gene expression and DNA methylation in breast, ovarian and endometrial cancers. **a** Pearson correlation between linear regression coefficient of age on gene expression or methylation from all samples and only samples without germline predisposition mutations. Pearson correlation coefficient r and *p*-value (two-sided test) are shown in the figures. **b** Overlap between age-DEGs or age-DMGs identified from all samples and only samples without germline predisposition mutations.

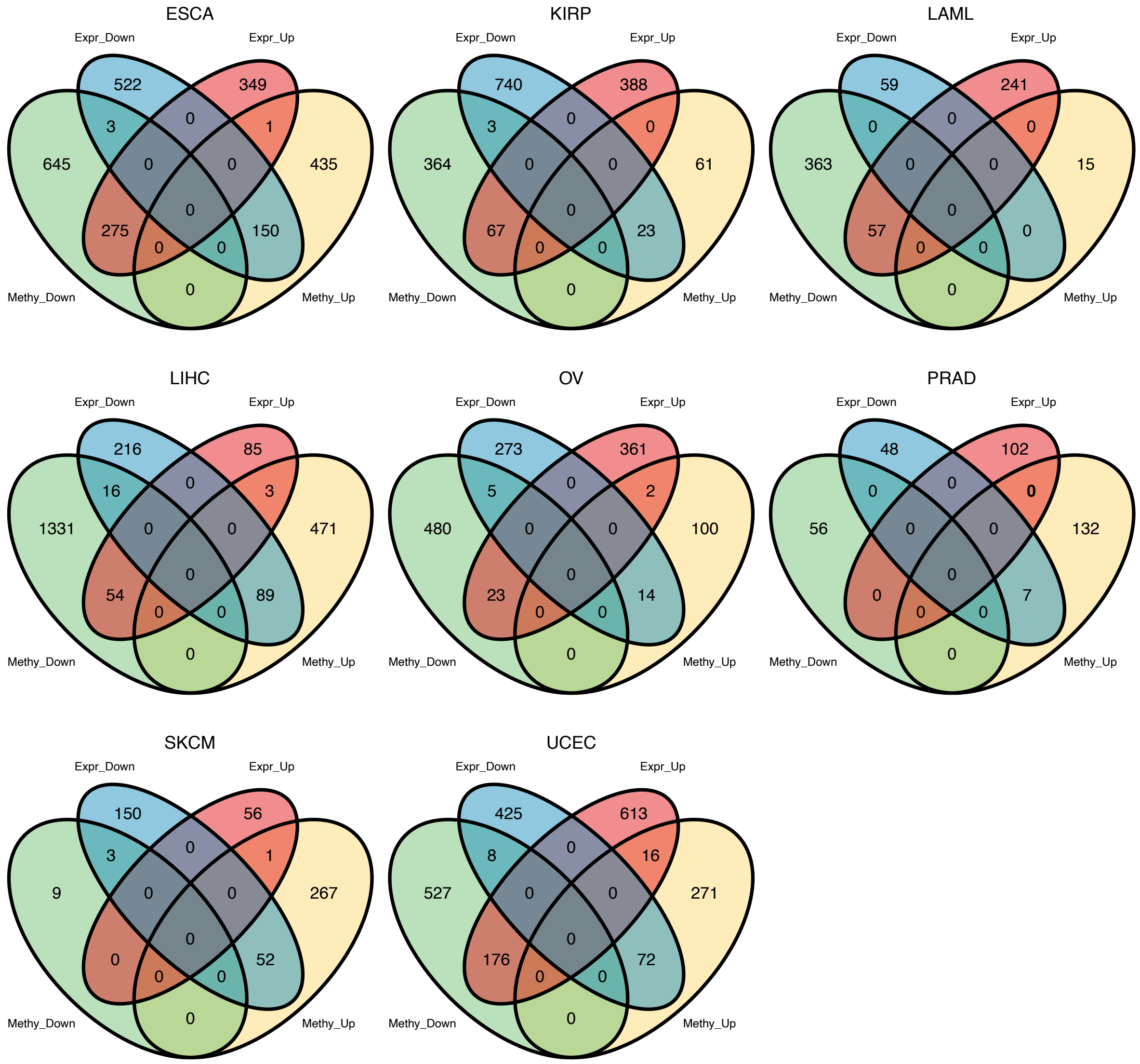

Supplementary Fig. 13

Venn diagrams of the overlap between age-DEGs and age-DMGs in 8 cancer types. Age-DEGs were separated into genes up-regulated with age (Expr_Up) and genes down-regulated with age (Expr_Down). Age-DMGs were classified into genes with increased methylation with age (Methy_Up) and genes with decreased methylation with age (Methy_Down).

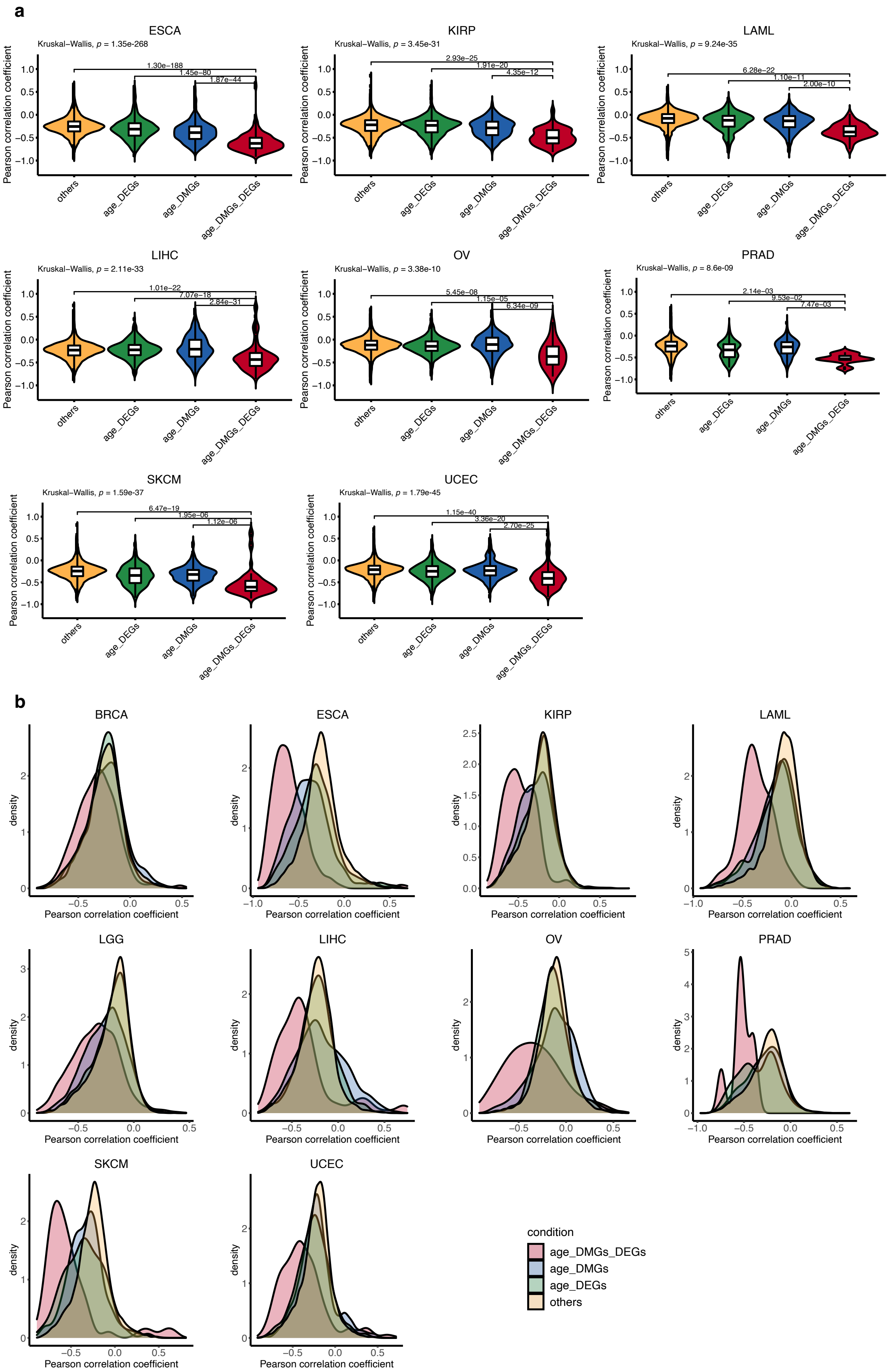
a
ESCA
Kruskal–Wallis, p = 1.35e-268
1.30e−188
1.45e−80
1.87e−44
KIRP
Kruskal–Wallis, p = 3.45e-31
2.93e−25
1.91e−20
4.35e−12
LAML
Kruskal–Wallis, p = 9.24e-35
6.28e−22
1.10e−11
2.00e−10
LIHC
Kruskal–Wallis, p = 2.11e-33
1.01e−22
7.07e−18
2.84e−31
OV
Kruskal–Wallis, p = 3.38e-10
5.45e−08
1.15e−05
6.34e−09
PRAD
Kruskal–Wallis, p = 8.6e-09
2.14e−03
9.53e−02
7.47e−03
SKCM
Kruskal–Wallis, p = 1.59e-37
6.47e−19
1.95e−06
1.12e−06
UCEC
Kruskal–Wallis, p = 1.79e-45
1.15e−40
3.36e−20
2.70e−25
Pearson correlation coefficient
others
age_DEGs
age_DMGs
age_DMGs_DEGs
b
BRCA
ESCA
KIRP
LAML
LGG
LIHC
OV
PRAD
SKCM
UCEC
density
Pearson correlation coefficient
condition
age_DMGs_DEGs
age_DMGs
age_DEGs
others

Supplementary Fig. 14

Pearson correlation coefficient between methylation and gene expression. **a** Violin plots showing the distribution of the Pearson correlation coefficient between methylation and gene expression in 8 cancer types. Genes were grouped into (1) overlapping genes between age-DMGs and age-DEGs (age-DMGs-DEGs), (2) age-DMGs only genes, (3) age-DEGs only genes, and (4) other genes. ESCA others n = 11564, age_DEGs n = 871, age_DMGs n = 1080, age_DMGs_DEGs n = 429 genes; KIRP others n = 11871, age_DEGs n = 1128, age_DMGs n = 425, age_DMGs_DEGs n = 93 genes; LAML others n = 9011, age_DEGs n = 300, age_DMGs n = 378, age_DMGs_DEGs n = 57 genes; LIHC others n = 10906, age_DEGs n = 1802, age_DMGs n = 301, age_DMGs_DEGs n = 162 genes; OV others n = 9222, age_DEGs n = 634, age_DMGs n = 580, age_DMGs_DEGs n = 44 genes; PRAD others n = 13365, age_DEGs n = 150, age_DMGs n = 188, age_DMGs_DEGs n = 7 genes; SKCM others n = 12786, age_DEGs n = 206, age_DMGs n = 276, age_DMGs_DEGs n = 56 genes; UCEC others n = 11618, age_DEGs n = 1038, age_DMGs n = 798, age_DMGs_DEGs n = 272 genes. The group comparison was performed by the Kruskal-Wallis test. The pairwise comparisons were done using two-sided Dunn's test. *P*-values from two-sided Dunn's test between age-DMGs-DEGs and the other groups are shown. The middle bar of the boxplot is the median. The box represents interquartile range (IQR), 25th to 75th percentile. Whiskers represent a distance of 1.5 x IQR. **b** Density plots showing the distribution of the Pearson correlation coefficient between methylation and gene expression, in four groups of genes, across 10 cancer types.

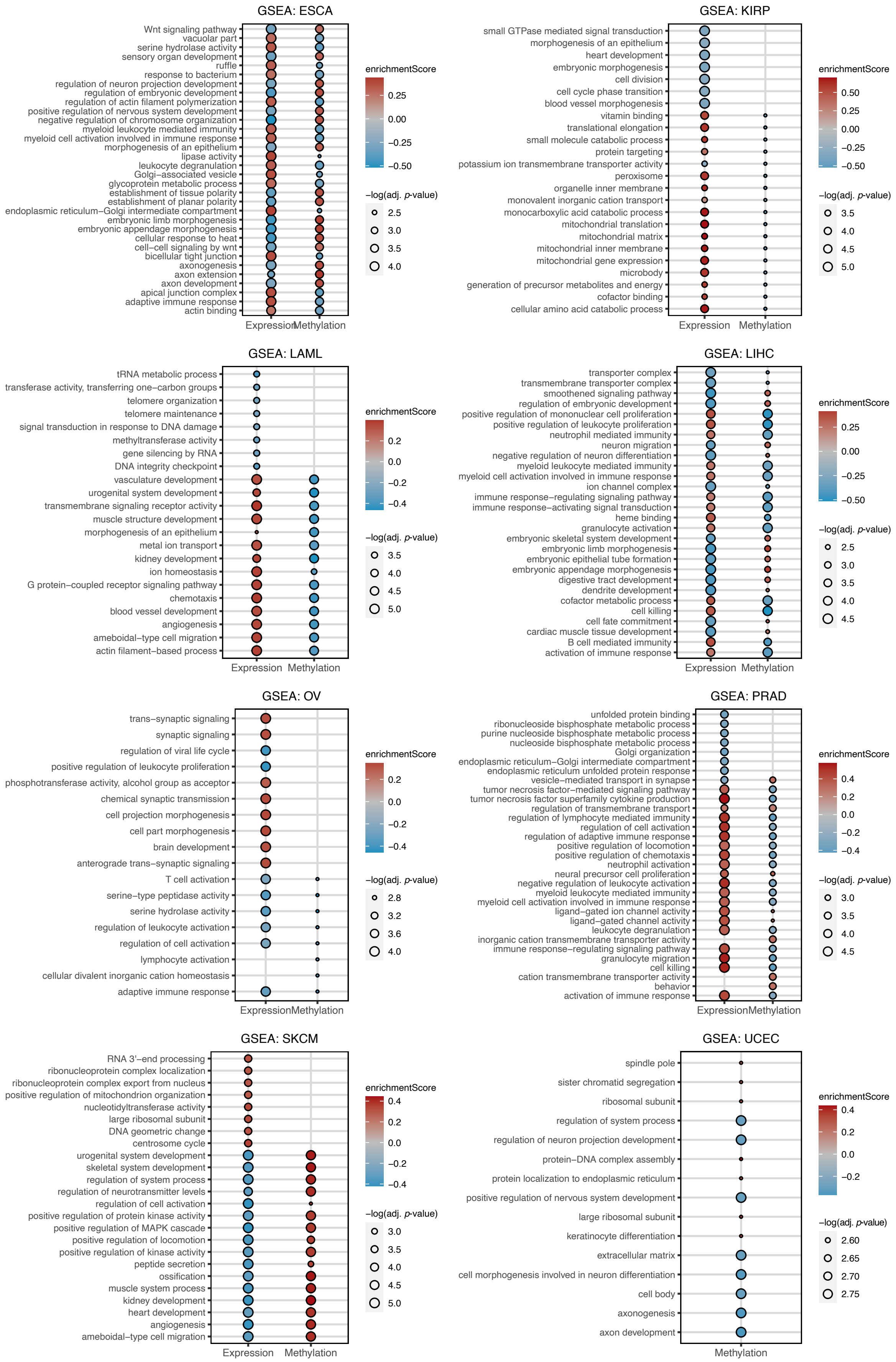
GSEA: ESCA
Wnt signaling pathway
vacuolar part
serine hydrolase activity
sensory organ development
ruffle
response to bacterium
regulation of neuron projection development
regulation of embryonic development
regulation of actin filament polymerization
positive regulation of nervous system development
negative regulation of chromosome organization
myeloid leukocyte mediated immunity
myeloid cell activation involved in immune response
morphogenesis of an epithelium
lipase activity
leukocyte degranulation
Golgi−associated vesicle
glycoprotein metabolic process
establishment of tissue polarity
establishment of planar polarity
endoplasmic reticulum−Golgi intermediate compartment
embryonic limb morphogenesis
embryonic appendage morphogenesis
cellular response to heat
cell−cell signaling by wnt
bicellular tight junction
axonogenesis
axon extension
axon development
apical junction complex
adaptive immune response
actin binding
Expression
Methylation
enrichmentScore
0.25
0.00
−0.25
−0.50
−log(adj. p-value)
2.5
3.0
3.5
4.0
GSEA: KIRP
small GTPase mediated signal transduction
morphogenesis of an epithelium
heart development
embryonic morphogenesis
cell division
cell cycle phase transition
blood vessel morphogenesis
vitamin binding
translational elongation
small molecule catabolic process
protein targeting
potassium ion transmembrane transporter activity
peroxisome
organelle inner membrane
monovalent inorganic cation transport
monocarboxylic acid catabolic process
mitochondrial translation
mitochondrial matrix
mitochondrial inner membrane
mitochondrial gene expression
microbody
generation of precursor metabolites and energy
cofactor binding
cellular amino acid catabolic process
Expression
Methylation
enrichmentScore
0.50
0.25
0.00
−0.25
−0.50
−log(adj. p-value)
3.5
4.0
4.5
5.0
GSEA: LAML
tRNA metabolic process
transferase activity, transferring one−carbon groups
telomere organization
telomere maintenance
signal transduction in response to DNA damage
methyltransferase activity
gene silencing by RNA
DNA integrity checkpoint
vasculature development
urogenital system development
transmembrane signaling receptor activity
muscle structure development
morphogenesis of an epithelium
metal ion transport
kidney development
ion homeostasis
G protein−coupled receptor signaling pathway
chemotaxis
blood vessel development
angiogenesis
ameboidal−type cell migration
actin filament−based process
Expression
Methylation
enrichmentScore
0.2
0.0
−0.2
−0.4
−log(adj. p-value)
3.5
4.0
4.5
5.0
GSEA: LIHC
transporter complex
transmembrane transporter complex
smoothened signaling pathway
regulation of embryonic development
positive regulation of mononuclear cell proliferation
positive regulation of leukocyte proliferation
neutrophil mediated immunity
neuron migration
negative regulation of neuron differentiation
myeloid leukocyte mediated immunity
myeloid cell activation involved in immune response
ion channel complex
immune response−regulating signaling pathway
immune response−activating signal transduction
heme binding
granulocyte activation
embryonic skeletal system development
embryonic limb morphogenesis
embryonic epithelial tube formation
embryonic appendage morphogenesis
digestive tract development
dendrite development
cofactor metabolic process
cell killing
cell fate commitment
cardiac muscle tissue development
B cell mediated immunity
activation of immune response
Expression
Methylation
enrichmentScore
0.25
0.00
−0.25
−0.50
−log(adj. p-value)
2.5
3.0
3.5
4.0
4.5
GSEA: OV
trans−synaptic signaling
synaptic signaling
regulation of viral life cycle
positive regulation of leukocyte proliferation
phosphotransferase activity, alcohol group as acceptor
chemical synaptic transmission
cell projection morphogenesis
cell part morphogenesis
brain development
anterograde trans−synaptic signaling
T cell activation
serine−type peptidase activity
serine hydrolase activity
regulation of leukocyte activation
regulation of cell activation
lymphocyte activation
cellular divalent inorganic cation homeostasis
adaptive immune response
Expression
Methylation
enrichmentScore
0.2
0.0
−0.2
−0.4
−log(adj. p-value)
2.8
3.2
3.6
4.0
GSEA: PRAD
unfolded protein binding
ribonucleoside bisphosphate metabolic process
purine nucleoside bisphosphate metabolic process
nucleoside bisphosphate metabolic process
Golgi organization
endoplasmic reticulum−Golgi intermediate compartment
endoplasmic reticulum unfolded protein response
vesicle−mediated transport in synapse
tumor necrosis factor−mediated signaling pathway
tumor necrosis factor superfamily cytokine production
regulation of transmembrane transport
regulation of lymphocyte mediated immunity
regulation of cell activation
regulation of adaptive immune response
positive regulation of locomotion
positive regulation of chemotaxis
neutrophil activation
neural precursor cell proliferation
negative regulation of leukocyte activation
myeloid leukocyte mediated immunity
myeloid cell activation involved in immune response
ligand−gated ion channel activity
ligand−gated channel activity
leukocyte degranulation
inorganic cation transmembrane transporter activity
immune response−regulating signaling pathway
granulocyte migration
cell killing
cation transmembrane transporter activity
behavior
activation of immune response
Expression
Methylation
enrichmentScore
0.4
0.2
0.0
−0.2
−0.4
−log(adj. p-value)
3.0
3.5
4.0
4.5
GSEA: SKCM
RNA 3'−end processing
ribonucleoprotein complex localization
ribonucleoprotein complex export from nucleus
positive regulation of mitochondrion organization
nucleotidyltransferase activity
large ribosomal subunit
DNA geometric change
centrosome cycle
urogenital system development
skeletal system development
regulation of system process
regulation of neurotransmitter levels
regulation of cell activation
positive regulation of protein kinase activity
positive regulation of MAPK cascade
positive regulation of locomotion
positive regulation of kinase activity
peptide secretion
ossification
muscle system process
kidney development
heart development
angiogenesis
ameboidal−type cell migration
Expression
Methylation
enrichmentScore
0.4
0.2
0.0
−0.2
−0.4
−log(adj. p-value)
3.0
3.5
4.0
4.5
5.0
GSEA: UCEC
spindle pole
sister chromatid segregation
ribosomal subunit
regulation of system process
regulation of neuron projection development
protein−DNA complex assembly
protein localization to endoplasmic reticulum
positive regulation of nervous system development
large ribosomal subunit
keratinocyte differentiation
extracellular matrix
cell morphogenesis involved in neuron differentiation
cell body
axonogenesis
axon development
Methylation
enrichmentScore
0.4
0.2
0.0
−0.2
−log(adj. p-value)
2.60
2.65
2.70
2.75

Supplementary Fig. 15

The enriched age-related gene ontology (GO) terms identified by Gene set enrichment analysis (GSEA) in 8 cancer types. The dot size corresponds to a significant level (permutation test). A GO term was considered significantly enriched if adj. p-value < 0.05 for gene expression and adj. p-value < 0.1 for methylation. Multiple hypothesis testing correction was done using Benjamini–Hochberg procedure. Colours represent enrichment scores, red denotes positive score (enriched in older patients), while blue signifies negative score (enriched in younger patients). No enriched term was identified from the gene expression data of endometrial cancer (UCEC).
