# Supplementary material for: An integrative analysis of the age-associated multi-omic landscape across cancers
Source: Nat Commun. 2021 Apr 20;12:2345. doi: 10.1038/s41467-021-22560-y (PMC8058097; doi:10.1038/s41467-021-22560-y)
Supplement: Supplementary file 3 — Description of Additional Supplementary Files [file 41467_2021_22560_MOESM3_ESM.pdf]

Description of Additional Supplementary Files

File Name: Supplementary Data 1
Description: Summary of the number of samples and clinical variables used in the study.

File Name: Supplementary Data 2
Description: Association between age and GI scores. Simple linear regression was performed to access the association between age and GI scores in Pan-cancer and each cancer type. Cancers with a significant association (adj. p-value < 0.05) were further investigated using multiple linear regression adjusting for clinical variables. Multiple hypothesis testing correction was done using Benjamini–Hochberg procedure.

File Name: Supplementary Data 3
Description: Description: Association between age and percent genomic LOH. Simple linear regression was performed to access the association between age and percent genomic LOH in Pan-cancer and each cancer type. Cancer types with a significant association (adj. p-value < 0.05) were further investigated using multiple linear regression adjusting for clinical variables. Multiple hypothesis testing correction was done using Benjamini–Hochberg procedure.

File Name: Supplementary Data 4
Description: Association between age and WGD. Simple logistic regression was performed to access the association between age and WGD in Pan-cancer and each cancer type. Cancer types with a significant association (adj. p-value < 0.05) were further investigated using multiple logistic regression adjusting for clinical variables. Multiple hypothesis testing correction was done using Benjamini–Hochberg procedure

File Name: Supplementary Data 5
Description: Association between age and SCNA scores. Overall, chromosome-level, arm-level and focal-level SCNA scores are provided in this supplementary data. The association between age and overall, chromosome/arm-level, and focal-level SCNA scores for each cancer type was investigated using simple linear regression. Cancer types with a significant association (adj. p-value < 0.05) were then subjected to multiple linear regression analysis adjusting for clinical variables. Multiple hypothesis testing correction was done using Benjamini–Hochberg procedure.

File Name: Supplementary Data 6
Description: Association between age and arm-level SCNAs. Simple logistic regression was performed to access the association between age and recurrently gain or loss arms in each cancer type. Chromosome arms with a significant association (adj. p-value < 0.05) were further investigated using multiple logistic regression adjusting for clinical variables. Multiple hypothesis testing correction was done using Benjamini–Hochberg procedure.

File Name: Supplementary Data 7
Description: Association between age and focal-level SCNAs. Simple logistic regression was performed to access the association between age and recurrently gain or loss regions in each cancer type. Regions with a significant association (adj. p-value < 0.05) were further investigated using multiple logistic regression adjusting for clinical variables. Multiple hypothesis testing correction was done using Benjamini–Hochberg procedure. Correlation between copy-number and gene expression was investigated using Pearson correlation (two-sided test).

File Name: Supplementary Data 8
Description: List of previously identified cancer driver genes.

File Name: Supplementary Data 9
Description: : Association between age and mutational burden. Simple linear regression was performed to access the association between age and mutational burden in each cancer type. Cancer types with a significant association (adj. p-value < 0.05) were further investigated using multiple linear regression adjusting for clinical variables. Multiple hypothesis testing correction was done using Benjamini–Hochberg procedure.

File Name: Supplementary Data 10
Description: Association between age and fraction contribution of mutation substitution classes. Simple linear regression was performed to access the association between age and each class of mutation substitution class in pan-cancer and each cancer type. Cancer types with a significant association (adj. p-value < 0.05) were further investigated using multiple linear regression adjusting for clinical variables. Multiple hypothesis testing correction was done using Benjamini–Hochberg procedure.

File Name: Supplementary Data 11
Description: Association between age and somatic mutations. Simple logistic regression was performed to access the association between age and somatic mutations in pan-cancer and each cancer type. Genes with a significant association between age and somatic mutations (adj. p-value < 0.05) were further investigated using multiple logistic regression adjusting for clinical variables. Multiple hypothesis testing correction was done using Benjamini–Hochberg procedure.

File Name: Supplementary Data 12
Description: Association between age and oncogenic signalling pathway. Simple logistic regression was performed to access the association between age and pathway alterations in pan-cancer and each cancer type. Cancer types with a significant association between age and pathway alterations (adj. p-value < 0.05) were further investigated using multiple logistic regression adjusting for clinical variables. Multiple hypothesis testing correction was done using Benjamini–Hochberg procedure.

File Name: Supplementary Data 13
Description: Gene expression changes with age. Normalised mRNA expression in RSEM for each TCGA cancer type was log2-transformed before subjected to the multiple linear regression analysis adjusting for clinical factors. Multiple hypothesis testing correction was done using Benjamini–Hochberg procedure. Genes with adj. p-value < 0.05 were considered significantly differentially expressed genes with age (age-DEGs).

File Name: Supplementary Data 14
Description: DNA methylation changes with age. DNA methylation data was presented as [illegible]-values, which are the ratio of the intensities of methylated and unmethylated alleles. we used the one-to-one mapping genes and probes by selecting the probes that are most negatively correlated with the corresponding gene expression multiple linear regression analysis adjusting for clinical factors. Multiple hypothesis testing correction was done using Benjamini–Hochberg procedure. Genes with adj. p-value < 0.05 were considered significantly differentially methylated genes with age (age-DMGs).

File Name: Supplementary Data 15
Description: Gene expression and DNA methylation changes with age in samples without germline mutations. Samples harbouring germline mutations in BRCA1, BRCA2 and TP53 were excluded from breast, ovarian and endometrial cancer cohorts. Multiple linear regression analysis adjusting for clinical factors were performed to access the association between age and gene expression/methylation in these three cancer types. Multiple hypothesis testing correction was done using Benjamini–Hochberg procedure. Genes with adj. p-value < 0.05 were considered significantly differentially expressed or differentially methylated genes with age (age-DEGs or age-DMGs).

File Name: Supplementary Data 16
Description: Number of overlapping genes between age-DEGs and age-DMGs.

File Name: Supplementary Data 17
Description: Comparison of the correlation coefficient between methylation and gene expression in (1) common genes between age-DMGs and age-DEGs (age-DMGs-DEGs), (2) age-DMGs only genes, (3) age-DEGs only genes, and (4) other genes. The correlation between gene expression and DNA methylation was calculated using Pearson correlation (two-sided test). Kruskal-Wallis test was used to investigate the differences between correlation coefficients among groups. The pairwise comparisons were carried out by two-sided Dunn's test.

File Name: Supplementary Data 18
Description: List of enriched GO terms identified using GSEA. Gene Set Enrichment Analysis (GSEA) was performed to investigate the Gene Ontology (GO) terms that are enriched in gene expression and methylation data from younger or older patients (permutation test). A GO term was considered significantly enriched if adj. p-value < 0.05 for gene expression and adj. p-value < 0.1 for methylation. Multiple hypothesis testing correction was done using Benjamini–Hochberg procedure.
