# Supplementary material for: An integrative analysis of the age-associated multi-omic landscape across cancers
Source: Nat Commun. 2021 Apr 20;12:2345. doi: 10.1038/s41467-021-22560-y (PMC8058097; doi:10.1038/s41467-021-22560-y)
Supplement: Supplementary file 22 — Reporting Summary [file 41467_2021_22560_MOESM22_ESM.pdf]

nature research

Corresponding author(s): João Pedro de Magalhães

Last updated by author(s): Feb 10, 2021

Reporting Summary

Nature Research wishes to improve the reproducibility of the work that we publish. This form provides structure for consistency and transparency in reporting. For further information on Nature Research policies, see our Editorial Policies and the Editorial Policy Checklist.

Statistics

For all statistical analyses, confirm that the following items are present in the figure legend, table legend, main text, or Methods section.

n/a	Confirmed	
☐	☒	The exact sample size (*n*) for each experimental group/condition, given as a discrete number and unit of measurement
☐	☒	A statement on whether measurements were taken from distinct samples or whether the same sample was measured repeatedly
☐	☒	The statistical test(s) used AND whether they are one- or two-sided<br>*Only common tests should be described solely by name; describe more complex techniques in the Methods section.*
☐	☒	A description of all covariates tested
☐	☒	A description of any assumptions or corrections, such as tests of normality and adjustment for multiple comparisons
☐	☒	A full description of the statistical parameters including central tendency (e.g. means) or other basic estimates (e.g. regression coefficient) AND variation (e.g. standard deviation) or associated estimates of uncertainty (e.g. confidence intervals)
☐	☒	For null hypothesis testing, the test statistic (e.g. *F*, *t*, *r*) with confidence intervals, effect sizes, degrees of freedom and *P* value noted<br>*Give P values as exact values whenever suitable.*
☒	☐	For Bayesian analysis, information on the choice of priors and Markov chain Monte Carlo settings
☒	☐	For hierarchical and complex designs, identification of the appropriate level for tests and full reporting of outcomes
☐	☒	Estimates of effect sizes (e.g. Cohen's *d*, Pearson's *r*), indicating how they were calculated

Our web collection on statistics for biologists contains articles on many of the points above.

Software and code

Policy information about availability of computer code

Data collection	No new data were collected in this study.
Data analysis	ASCAT (version 2.4.2) and GISTIC2.0 (version 2.0.22) were used for genomic analysis. All statistical analyses were carried out using R (version 3.6.3). Packages used for data analysis included TCGAbiolinks (version 2.14.1), logistf (version 1.23), maftools (version 3.3.2), biomaRt (version 2.46.0, data based on Ensembl version 100, April 2020), ClusterProfiler (version 3.14.3). Plots were generated using ggplot2 (version 3.3.2), ggrepel (version 0.8.2), ggpubr (version 0.4.0), ComplexHeatmap (version 2.2.0), and VennDiagram (version 1.6.20). The custom scripts for data analysis and generate figures are available at https://github.com/maglab/Age-associated_cancer_genome.

For manuscripts utilizing custom algorithms or software that are central to the research but not yet described in published literature, software must be made available to editors and reviewers. We strongly encourage code deposition in a community repository (e.g. GitHub). See the Nature Research guidelines for submitting code & software for further information.

Data

Policy information about availability of data

All manuscripts must include a data availability statement. This statement should provide the following information, where applicable:

- Accession codes, unique identifiers, or web links for publicly available datasets
- A list of figures that have associated raw data
- A description of any restrictions on data availability

We used publicly available data provided by The Cancer Genome Atlas (TCGA). The data are publicly available and can be downloaded from NCI's Genomic Data Commons portal (https://portal.gdc.cancer.gov/), TCGAbiolinks (version 2.14.1) and Broad GDAC Firehose (http://gdac.broadinstitute.org/). The mutation annotation format (MAF) file was downloaded from the TCGA MC3 project (https://gdc.cancer.gov/about-data/publications/mc3-2017). List of known cancer driver genes were compiled from COSMIC database version 91 (https://cancer.sanger.ac.uk/cosmic), Lawrence et al. (https://doi.org/10.1038/nature12912) and TCGA

Pan-Cancer study (https://doi.org/10.1016/j.cell.2018.02.060). Oncogenic signalling pathway data was obtained from Sanchez-Vega et al (https://doi.org/10.1016/j.cell.2018.03.035). Allele-specific copy number, tumour ploidy, tumour purity, GI scores and WGD status of TCGA tumours generated by ASCAT (version 2.4.2) are available at https://github.com/Crick-CancerGenomics/ascat/tree/master/ReleasedData/TCGA_SNP6_hg19. Source data are provided with this paper. The remaining data are available within the Article, Supplementary Information or available from the authors upon request.

Field-specific reporting

Please select the one below that is the best fit for your research. If you are not sure, read the appropriate sections before making your selection.

- [x] Life sciences
- [ ] Behavioural & social sciences
- [ ] Ecological, evolutionary & environmental sciences

For a reference copy of the document with all sections, see nature.com/documents/nr-reporting-summary-flat.pdf

Life sciences study design

All studies must disclose on these points even when the disclosure is negative.

Sample size	Sample sizes were not predetermined as we aimed to use all samples available on TCGA that can be unambiguously assigned allele-specific copy number, tumour ploidy and tumour purity using ASCAT. Number of samples in each analysis can be found in the Supplementary Data 1.
Data exclusions	From all available TCGA samples, we limited our analyses to samples that can be unambiguously assigned allele-specific copy number, tumour ploidy and tumour purity using ASCAT. The rationale behind this exclusion is that we included in our analysis model the tumour purity. In somatic mutation analysis, we excluded samples that contain more than 1,000 SNVs per exome (hypermutated tumours) and that are MSI-H tumours, as described in the method section. The rationale behind this exclusion is to obtain the results from age-associated mutations that are not confounded by the present of hypermutated tumours.
Replication	Replication is not applicable, since there was no experimental data.
Randomization	Randomization was not relevant to the study. There was no treatment and control groups.
Blinding	Blinding was not relevant to the study. There was no treatment and control groups.

Reporting for specific materials, systems and methods

We require information from authors about some types of materials, experimental systems and methods used in many studies. Here, indicate whether each material, system or method listed is relevant to your study. If you are not sure if a list item applies to your research, read the appropriate section before selecting a response.

Materials & experimental systems

n/a	Involved in the study	
x		Antibodies
x		Eukaryotic cell lines
x		Palaeontology and archaeology
x		Animals and other organisms
x		Human research participants
x		Clinical data
x		Dual use research of concern

Methods

n/a	Involved in the study	
x		ChIP-seq
x		Flow cytometry
x		MRI-based neuroimaging
